# An Anti-CD34 Antibody-Functionalized Clinical-Grade POSS-PCU Nanocomposite Polymer for Cardiovascular Stent Coating Applications: A Preliminary Assessment of Endothelial Progenitor Cell Capture and Hemocompatibility

**DOI:** 10.1371/journal.pone.0077112

**Published:** 2013-10-08

**Authors:** Aaron Tan, Debbie Goh, Yasmin Farhatnia, Natasha G, Jing Lim, Swee-Hin Teoh, Jayakumar Rajadas, Mohammad S. Alavijeh, Alexander M. Seifalian

**Affiliations:** 1 Centre for Nanotechnology and Regenerative Medicine, UCL Division of Surgery & Interventional Science, University College London, London, United Kingdom; 2 UCL Medical School, University College London, London, United Kingdom; 3 Division of Bioengineering, School of Chemical and Biomedical Engineering, Nanyang Technological University, Singapore, Singapore; 4 Biomaterials and Advanced Drug Delivery Laboratory, School of Medicine, Stanford University, Stanford, California, United States of America; 5 Pharmidex Pharmaceutical Services Ltd., London, United Kingdom; 6 Royal Free London NHS Foundation Trust, London, United Kingdom; Université de Technologie de Compiègne, France

## Abstract

*In situ* endothelialization of cardiovascular implants has emerged in recent years as an attractive means of targeting the persistent problems of thrombosis and intimal hyperplasia. This study aimed to investigate the efficacy of immobilizing anti-CD34 antibodies onto a POSS-PCU nanocomposite polymer surface to sequester endothelial progenitor cells (EPCs) from human blood, and to characterize the surface properties and hemocompatibility of this surface. Amine-functionalized fumed silica was used to covalently conjugate anti-CD34 to the polymer surface. Water contact angle, fluorescence microscopy, and scanning electron microscopy were used for surface characterization. Peripheral blood mononuclear cells (PBMCs) were seeded on modified and pristine POSS-PCU polymer films. After 7 days, adhered cells were immunostained for the expression of EPC and endothelial cell markers, and assessed for the formation of EPC colonies. Hemocompatibility was assessed by thromboelastography, and platelet activation and adhesion assays. The number of EPC colonies formed on anti-CD34-coated POSS-PCU surfaces was not significantly higher than that of POSS-PCU (5.0±1.0 vs. 1.7±0.6, *p*>0.05). However, antibody conjugation significantly improved hemocompatibility, as seen from the prolonged reaction and clotting times, decreased angle and maximum amplitude (*p*<0.05), as well as decreased platelet adhesion (76.8±7.8 vs. 8.4±0.7, *p*<0.05) and activation. Here, we demonstrate that POSS-PCU surface immobilized anti-CD34 antibodies selectively captured CD34^+^ cells from peripheral blood, although only a minority of these were EPCs. Nevertheless, antibody conjugation significantly improves the hemocompatibility of POSS-PCU, and should therefore continue to be explored in combination with other strategies to improve the specificity of EPC capture to promote *in situ* endothelialization.

## Introduction

With the rapid advancement of interventional cardiology over the past decade, percutaneous coronary intervention (PCI) has become the treatment of choice for atherosclerotic coronary artery disease (CAD) [1]. Although PCI is widely viewed as an acceptable alternative to coronary artery bypass graft (CABG) surgery, the recent SYNTAX trial revealed that CABG should remain the standard of care for patients with complex lesions [2]. Nevertheless, the prevalence of PCI as a treatment for CAD warrants attention, especially in the realm of cardiovascular regenerative medicine. Majority of PCI involves plaque compression by balloon angioplasty, followed by the deployment of a stent, which acts as a permanent scaffold ensuring vessel patency. Early bare metal stents (BMS) were developed to limit post-angioplasty restenosis, although their success was limited by significant rates (15-30%) of in-stent restenosis (ISR) [3]. ISR is primarily a consequence of neointimal hyperplasia, which is a result of the body mounting an immunological response to the metal stent, as well as local mechanical vascular injury caused by stent deployment. Upregulation of inflammatory mediators and induction of thrombogenic cascades culminate in abnormal vascular smooth muscle cell (VSMC) proliferation, smooth muscle hypertrophy and extracellular matrix deposition, which result in luminal narrowing and vessel re-occlusion [4-8]. The introduction of polymer-coated drug-eluting stents (DES), which allow for localised delivery of anti-proliferative drugs such as sirolimus and paclitaxel to the neointima, was a key advance that resulted in dramatically reduced ISR rates of less than 10% in initial clinical trials. DES have thus become the standard of care and are used in over 85% of PCI [9].

Despite the clear short- to mid-term benefits of using DES over BMS, there have been concerns over the long-term safety of DES. Indeed, a recent meta-analysis in the Cochrane Review revealed that there were not statistically significant difference in death, acute myocardial infarction (MI), or thrombosis rates when comparing DES to BMS [10]. Furthermore, follow-up studies of patients who received first-generation DES (sirolimus- and paclitaxel-eluting stents) have revealed an association with increased cumulative incidence of very late (i.e., >1 year post-stenting) stent thrombosis (ST) [6]. ST is a life-threatening event, with mortality rates of up to 30%, and is postulated to be a result of non-selective drug inhibition of both endothelial cell (EC) and VSMC proliferation, which delay endothelial recovery [3,4]. Furthermore, an inflammatory reaction is induced by the polymer coating in which the drugs are dissolved in, and can cause delayed neointimal hyperplasia and restenosis. Moreover, to decrease the persistent risk of very late ST, long-term (6-12 months post-stenting) dual anti-platelet therapy (aspirin and clopidogrel) is required, and this in itself may bring about undesirable side effects such as hemorrhagic complications and thrombotic thrombocytopenic purpura. As rates of late ST remain higher with DES, the actual long-term benefits of DES over BMS have been called into question [11-15]. There is therefore an urgent need to develop new methods of circumventing both the problems of ISR and thrombosis seen in BMS and DES, for which endothelialization of the stent surface has emerged as a promising approach.

A hemocompatible polymer stent coating is therefore a crucial element, as while it must act as a protective coating for the bare metal surface to prevent the problem of ISR as seen with BMS, it must not evoke an immunological hypersensitive response and subsequent stent thrombosis, which is the unaddressed problem currently seen with DES. This would carry with it the additional benefit of shortening or even doing away with the need for long-term dual anti-platelet therapy, which is currently the case with DES [16].

We have developed and patented a proprietary nanocomposite polymer, polyhedral oligomeric silsesquioxane-poly(carbonate-urea) urethane (POSS-PCU), to meet the need for functional nanomaterials for biomedical applications. The introduction of inert nano-sized POSS moieties into PCU has been shown to greatly enhance the mechanical, physical, and thermal properties of PCU, such as tensile strength, viscoelasticity, chemical stability, and calcification resistance [17-20]. Further characterisation of POSS-PCU in numerous studies has demonstrated that it is non-biodegradable, biocompatible and non-toxic, and is exceptionally anti-inflammatory and anti-thrombogenic *in vivo* compared to PTFE and PCU, evoking negligible immunoreactivity [17,20-23]. Additionally, it can be produced rapidly and in a clinically-appropriate time frame [24]. Its superior properties have allowed it to be used in a wide range of applications – it has been used in the bioengineering of a first-in-man artificial tracheobronchial implant [24], lacrimal drainage conduit [19], vascular bypass graft [25,26], as well as heart valves [27], and is currently being tested and optimised for coating stents.

For POSS-PCU to be a favourable platform for EPCs to specifically adhere to and proliferate on, biofunctionalization of POSS-PCU through the incorporation of EPC-specific antibodies such as anti-CD34 is necessary to achieve *in situ* endothelialization, which would then theoretically increase the probability of EPC capture and minimize the risk of VSMC proliferation. Ideally, stable integration of antibodies into POSS-PCU in the correct orientation for target cell-binding should be achieved without any significant detrimental effects on the physical properties and surface chemistry of POSS-PCU. To this end, de Mel (2011) investigated the possibility of incorporating amine-functionalized fumed silica (FS) into POSS-PCU to act as a nano-anchor for antibodies and peptides, and successfully demonstrated that the addition of amine-functionalized FS was able to facilitate antibody conjugation without negatively impacting the physical and biological properties of POSS-PCU [28].

**Figure 1:**
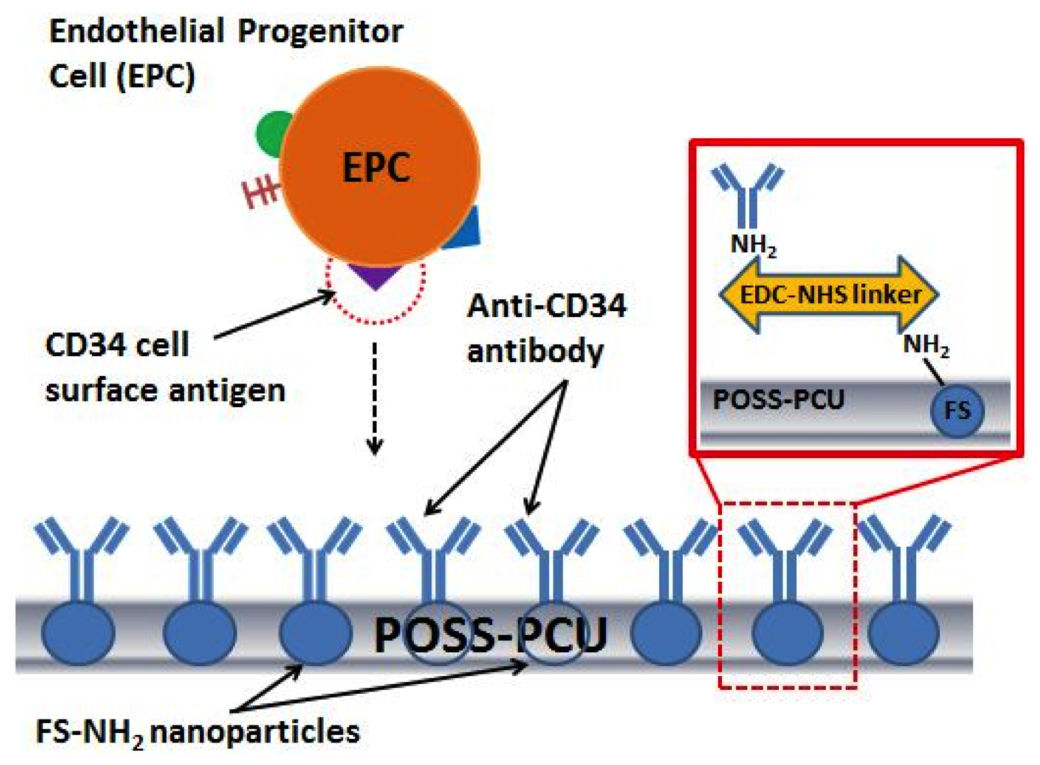
Anti-CD34 antibody immobilization on POSS-PCU polymer surface via amine-functionalized fumed silica nanoparticles and EDC-NHS linker. This method could represent a novel way of attracting circulating endothelial progenitor cells for accelerated endothelialization.

To covalently immobilize antibodies on the FS-modified POSS-PCU surface (POSS-PCU-FS) in a stable manner and in the correct orientation, EDC-NHS (N-ethyl-N’-(3-dimethylaminopropyl)-carbodiimide, N-hydroxy-succinimide) was chosen as a chemical linker between the NH_2_ groups in POSS-PCU-FS and the NH_2_ groups on the Fc region of antibodies (Figure 1). This is necessary for ensuring a stable antibody coating, as preliminary studies using an ultrasonic spray atomizer (MediCoat DES 1000, Sono-Tek Corporation, USA) to spray antibodies onto POSS-PCU-FS were unsuccessful in producing a stable, uniform antibody layer (data not shown). Furthermore, studies have shown that random antibody immobilization lowers the proportion of sites available for antigen-binding, whereas immobilizing antibodies in the correct orientation increases antigen-binding activity and efficiency [29,30].

As such, this *in vitro* study aimed to characterise the anti-CD34 biofunctionalized POSS-PCU surface, and assess its ability to both enhance EPC capture from peripheral blood as well as increase hemocompatibility.

## Materials and Methods

All chemical reagents were purchased from Sigma-Aldrich UK, and cell culture reagents were purchased from Life Technologies (Invitrogen) UK, unless otherwise stated. All experiments were conducted in triplicates (n=3) unless otherwise stated.

### POSS-PCU polymer synthesis

The synthesis of our proprietary POSS-PCU has been described in detail elsewhere [21]. Briefly, a mixture of polycarbonate diol (2000 mwt) and trans-cyclohexanechlorohydrinisobutyl-silsesquioxane (POSS, Hybrid Plastics Inc) was placed in a reaction flask equipped with a mechanical stirrer and nitrogen inlet, heated to 135°C in order to dissolve the POSS cage in polycarbonate diol, and finally cooled to 70°C. 4,4’-methylene-bis-(phenyl isocyanate) was then added to the mixture to form a pre-polymer. Dimethylacetamide (DMAc) was subsequently added slowly to dissolve the pre-polymer, forming a solution that was then cooled to 40°C. Following solvation, chain extension of the pre-polymer solution was achieved by drop-wise addition of a mixture of ethylenediamine and diethylamine (in DMAc) to form a final polymer solution of POSS-modified polycarbonate urea-urethane (in DMAc).

### Amine-functionalization of fumed silica

In-depth discussion about amine-functionalization of fumed silica was developed in our lab by de Mel, and is described elsewhere [28]. Briefly, functionalization of fumed silica (Aerosil® A200 fumed silica, Evonik Industries) with amine (NH_2_) groups was carried out as follows. 1g of A200 fumed silica was first mixed with 100 mL of propan-2-ol, 5mL of 3-aminopropyltriethoxysilane (APTES), and 5mL of deionised water in a pre-silanised round-bottomed flask. This mixture was sonicated in a bath sonicator (Grant Instruments, UK) for 45min. To prevent overheating, the flask containing the mixture was cooled in ice, and the water in the sonicator was changed every 15min. Subsequently, the resultant mixture was refluxed at 70 °C for 6hrs. Following which, the mixture was centrifugated at 4000rpm for 40min. The resultant supernatant was discarded, while the pellet was re-suspended in propan-2-ol and centrifugated again. This step was repeated 5 times. Finally, the pellet was placed in a heating oven (Binder GmbH) at 65 °C for 24hrs. The resultant amine-functionalized fumed silica (FS-NH_2_) had a powdery, white appearance.

### Incorporation of amine-functionalized fumed silica into POSS-PCU

FS-NH_2_ incorporation was developed in our lab by de Mel, and is described elsewhere [28]. Briefly, FS-NH_2_ nanoparticles were incorporated into POSS-PCU (18% in DMAc) to make up a POSS-PCU-FS mixture (2% FS-NH_2_ in 18% POSS-PCU) as follows. First, FS-NH_2_ was added to DMAc in a glass scintillation vial, and dissolving by sonication for 30min at room temperature in a bath sonicator. The dissolved FS-NH_2_ was then added to 18% POSS-PCU and subjected to further mixing with an Ultrasonic Processor (Cole-Parmer, UK) for three 20s cycles. The mixture was left to stand for an hour in order to allow air bubbles to dissolve.

### POSS-PCU and POSS-PCU-FS polymer sheet preparation

Thin POSS-PCU and POSS-PCU-FS polymer sheets were made by casting POSS-PCU and POSS-PCU-FS polymer mixtures on separate 10cm by 10cm stainless steel plates and leaving them to dry in a heating oven 65°C for 24hrs. Following which, polymer sheets were peeled off and cut into discs (16mm diameter, 2cm^2^ surface area) using a cutting press and die (Wallace Instruments, UK), in order to fit 24-well plates. The discs were then autoclaved before antibody conjugation where necessary. Preliminary studies have shown that autoclaving does not negatively affect antibody conjugation to polymer (data not shown).

### Covalent immobilization of anti-CD34 antibody on POSS-PCU-FS discs

Covalent immobilization of antibodies/peptide motifs onto POSS-PCU was developed by del Mel in our lab, and in-depth discussion is described elsewhere [28]. Briefly, N-ethyl-N’-(3-dimethylaminopropyl)carbodiimide hydrochloride (EDC) (0.4mg/mL), N-hydroxy-succinimide (NHS) (0.575mg/mL), and succinic acid (2.5 mg/mL) were weighted and dissolved in phosphate-buffered saline (PBS) to make up EDC-NHS. EDC-NHS was then placed on a roller mixer (Stuart Equipment) for 1hr before being filter-sterilised in a laminar flow hood.

EDC-NHS activation of POSS-PCU-FS surfaces was carried out by placing autoclaved POSS-PCU-FS discs in a 24-well plate under sterile conditions, and incubating the discs with EDC-NHS for 30min at room temperature in the dark on a Luckham R100 Rotatest shaker (Richmond Scientific Ltd.). Subsequently, the discs were washed with PBS to remove any unreacted functionalities.

Mouse anti-human CD34 and mouse anti-human IgG antibodies were both separately diluted in 0.1% PBS-Tween 20 (PBST) to a final concentration of 2µg/mL, which gives a surface coating density of 1µg/cm^2^ for a 2cm^2^ polymer disc. The diluted antibody solutions were then filter-sterilised in a laminar flow hood. Either anti-CD34 or anti-IgG antibody dilutions were added to their respective EDC-NHS-activated POSS-PCU-FS discs, and placed on the shaker for 30min in the dark at room temperature. The discs were then transferred to a 4°C fridge and incubated overnight in the dark to facilitate covalent immobilization of antibodies. Subsequently, discs were washed with PBS to remove non-specifically adsorbed antibodies.

### Scanning electron microscopy

Surface morphology of POSS-PCU, POSS-PCU-FS, and anti-CD34-coated POSS-PCU-FS (POSS-PCU-FS+CD34) was assessed using scanning electron microscopy (SEM) (FEI, Quanta 200 SEM). Samples were first given 20nm gold coating in a Quroum Q150T Sputter Coater using argon prior to SEM imaging.

### Stability assay for immobilized antibody coating

Evaluation of the presence of immobilized anti-CD34 as well as the uniformity and stability of this antibody coating over time was assessed by immunostaining with Quantum Dot (QD)-anti-IgG conjugates. POSS-PCU-FS+CD34 discs were first washed in deionised water, then immersed in PBS and left on the shaker at room temperature in the dark. After 24hrs and 72hrs, discs were removed and incubated with red QD-anti-IgG (goat anti-mouse) conjugates for detection of immobilized mouse anti-CD34. Fluorescent micrographs were taken, and the fluorescence intensities of QD-anti-IgG immunostained POSS-PCU-FS+CD34 surfaces after 24 and 72hrs (n=3 per time point) were quantified using ImageJ (NIH) software. Unmodified POSS-PCU and POSS-PCU-FS were used as controls.

### Water Contact Angle

Water contact angle (WCA) measurements were performed for POSS-PCU, POSS-PCU-FS, and POSS-PCU-FS+CD34 films with a KRÜSS DSA 100 machine and Drop Shape Analysis software (EasyDrop DSA20E, Kruss) to compare the differences in surface wettability. The sessile drop method was used, with droplet volume and dispensing rate kept constant at 5.0µl and 195.1µl/min respectively. A total of 6 measurements were taken randomly at different spots of each disc (n=6), each within 10s of dispensing. Surfaces with WCA measurements of > 90° are considered hydrophobic, whereas those with angles < 90° are considered hydrophilic.

### Cell culture

All procedures that involved extraction of human blood via venipuncture were obtained from healthy volunteers, with informed consent, and approved by the Institutional Review Board (IRB) at the Division of Surgery and Interventional Science at University College London and Royal Free London NHS Foundation Trust.

20mL blood samples were collected with informed consent (Royal Free London NHS Foundation Trust Institutional Review Board (IRB)) from healthy adult human volunteers by venepuncture in ethylenediaminetetraacetic acid (EDTA) BD Vacutainer®. Samples were thoroughly mixed with the EDTA to prevent coagulation.

EPC isolation was carried out within an hour of blood sample collection, using a method that has been reported previously to produce EPC colonies that are able to differentiate into cells with EC markers and characteristics [31]. First, density gradient centrifugation was carried out by layering the blood sample over Histopaque-1077, and centrifuging in a MSE Mistral 3000i centrifuge at 400*g* for 30min at room temperature. The buffy coat layer containing the mononuclear fraction of peripheral blood, including EPCs, was carefully harvested and washed with Hank’s Balanced Salt Solution (HBSS) by centrifuging at 250*g* for 10min, discarding the supernatant, and re-suspending the pellet in HBSS. After 2 rounds of washing, isolated PBMCs were re-suspended in cell culture media (CCM), which comprised Medium 199 supplemented with 20% FBS, 1% glutamine, and 1% penicillin/streptomycin (all from Invitrogen). PBMCs were then enumerated using a hemocytometer and Trypan Blue exclusion dye, and then diluted in CCM to a final concentration of 1.0x10^6^ PBMCs/mL.

For EPC characterization, 2–3 x 10^6^ PBMCs/well were seeded in 6-well plates. After 3 days of culture, non-adherent cells were removed by gentle washing with PBS, while adherent cells were kept in culture for further cultivation. On days 5 and 7 post-seeding, half of the media in each well was carefully replaced with fresh CCM. The cell cultures were visualised every 2-3 days under light microscopy, and any eventful changes in cell morphology were recorded. After 7 days, cells were fixed and directly immunostained with QD-antibody conjugates, for the expression of CD34, VEGFR-2, CD31 (or PECAM-1, platelet endothelial cell adhesion molecule-1), and von Willebrand factor (vWF).

For the EPC capture assay, sterile anti-CD34-coated POSS-PCU-FS, mouse IgG isotype-coated POSS-PCU-FS (negative control), as well as unmodified POSS-PCU and POSS-PCU-FS discs, were placed in a 24-well plate and equilibrated in sterile CCM in a 37 ^o^C/5% CO_2_ incubator overnight. The next day, the CCM in each well was replaced by 1mL of 1x10^6^ PBMCs/mL PBMC suspension, and the plate was returned to the 37 ^o^C/5% CO_2_ incubator. For positive controls, PBMCs were seeded in empty wells, whereas POSS-PCU-FS discs with no cells seeded were used as negative controls. After 3 days of culture, non-adherent cells were removed by gentle washing with PBS, while adherent cells were kept in culture for further cultivation. On days 5 and 7 post-seeding, half of the media in each well was carefully replaced with fresh CCM. At day 7, cells were fixed and double-stained for EPC markers (CD34 and VEGFR-2) as well as EC markers (CD31 and vWF). In addition, the total number of EPC colonies formed per 10^6^ PBMCs seeded in each well was enumerated manually. EPC colonies were defined morphologically as central clusters of rounded cells with spindle-shaped cells emanating from the centre, which also stained positive for both CD34 and VEGFR-2 under a laser scanning confocal microscope [32-34].

### Immunostaining with quantum dot-antibody conjugates

Prior to direct immunostaining with QD-antibody conjugates, cells cultured on the various surfaces were first washed twice with 0.1% PBST, fixed with 2% paraformaldehyde (PFA, in 0.1% PBST) for 20min, then washed thrice with 0.1% PBST at room temperature. Cells were subsequently permeabilized in 0.5% Tween-20 (in PBS) for 15min, then washed thrice with 0.1% PBST.

The fixed cells were first blocked with 1% bovine serum albumin (BSA, in 0.1% PBST) for ½hr at 4°C in the dark, and then washed with 0.1% PBST before incubation with QD-antibody conjugates for 2hrs at 4°C in the dark. Samples were subsequently washed with 0.1% PBST, incubated with 4',6-diamidino-2-phenylindole (DAPI, 5µg/mL) for 5min at room temperature, washed with 0.1% PBST, and left in 0.1% PBST for imaging. Images were captured by a Nikon Eclipse TE300 inverted microscope equipped with an EZ-C1 confocal microscopy system (Nikon). A 488nm He-Ne excitation laser was used, and images were acquired using either a 515-530nm emission range channel (for green and blue fluorescence detection), or a 650LP channel (for red fluorescence detection).

Anti-CD34 (241µg/mL), anti-VEGFR-2 (250µg/mL), anti-vWF (200µg/mL; Santa Cruz), and anti-CD31 (500µg/mL) antibodies were conjugated with either red or green quantum dots and diluted in 1% BSA to make up 0.25µg/mL QD-antibody working solutions. VEGFR-2 and CD34 are EPC markers, whereas vWF and CD31 are EC markers. Hence, the following pairings of QD-antibodies were used: QD-anti-VEGFR-2 (red, emission maximum 640nm) and QD-anti-CD34 (green, emission maximum 555nm); QD-anti-vWF (red) and anti-CD31 (green). Additionally, cells were stained with blue DAPI nuclei counterstain.

### Thromboelastography (TEG)

Thrombelastography (TEG) was carried out using the TEG coagulation analyzer (TEG 5000® Thrombelastograph Haemostasis System) to evaluate contact activation and blood clotting kinetics of the anti-CD34-coated POSS-PCU-FS surface. TEG continuously monitors the entire clotting process and generates parameters that relate to each phase.

Standard TEG cups were coated with a thin, uniform layer of POSS-PCU-FS polymer (2% FS-NH_2_ in 18% POSS-PCU) and incubated upside down to dry in a 65°C oven overnight. These POSS-PCU-FS-coated cups were then incubated with EDC-NHS for 30min, washed with PBS, then incubated with anti-CD34 antibody solution (2µg/mL) overnight at 4°C. Anti-CD34 coated TEG cups were air-dried in a laminar flow hood prior to TEG analysis.

For each round of analysis, the TEG system was calibrated to 37°C before mounting anti-CD34 coated TEG cups in the TEG analyser. 20µL of 0.2M calcium chloride solution, a known initiator of blood coagulation, was added to each cup, followed by 340µL of citrated blood. Similar TEG analyses were performed on POSS-PCU-coated and POSS-PCU-FS-coated cups for comparison. Standard TEG cups with citrated whole blood (340µL) were used as positive controls, whereas standard cups containing citrated blood (300µL) mixed with 0.01M L-arginine (40µL) were used as negative controls, as L-arginine is known to have anti-coagulant effects on native whole blood [35]. To reduce variability due to blood samples, each test was repeated three times with blood drawn from the same volunteer at the same time.

### Platelet adhesion and activation assay

Sterile POSS-PCU, POSS-PCU-FS, and anti-CD34 coated POSS-PCU-FS discs were equilibrated overnight in sterile PBS in a 24-well plate placed in a 37 ^o^C/5% CO_2_ incubator. For the isolation of platelet-rich plasma (PRP) by density centrifugation, a 20mL blood sample was first collected from a healthy volunteer using BD Vacutainer® containing 0.109M buffered sodium citrate, and mixed gently. The citrated blood was carefully layered onto 20mL of Histopaque-1077 and centrifuged at 200*g* for 30min. The topmost clear yellow layer making up the PRP was carefully harvested. Average platelet number was determined by making 3 reference counts with a hemocytometer, following which, the harvested PRP was diluted in sterile PBS to give a final working concentration of 1x10^6^ platelets/mL.

PBS in each well was replaced with 1mL of diluted PRP (1x10^6^ platelets/mL), and incubated for 1 hour in a 37°C/5% CO_2_ incubator. After 1 hour, the supernatant in each well was gently transferred to separate Eppendorf tubes, and the number of platelets enumerated immediately using a hemocytometer.

The degree of platelet adhesion to each sample was expressed quantitatively as a Platelet Adhesion Index (PAI), which was calculated as follows:

$$\text{PAI (\%) = }\frac{\text{100 (}P_{i}-P_{s})}{P_{i}}$$

where P_i_ is the initial number of platelets seeded on each sample (10^6^), and P_s_ is the number remaining in the supernatant after the 1 hour incubation. A staging for platelet activation was previously described by Ko and Cooper (1993) [36], and is summarized in Table 1.

**Table 1:** The various stages of platelet activation. Adapted from Ko and Cooper 1993, ref [36].

**Type**	**Stage**	**Morphology**
**I**	Round	Round or spherical without evidence of pseudopodia
**II**	Dendritic	Round with early-stage pseudopodia without flattening
**III**	Spread-Dendritic	Pseudopodia at intermediate stage, with evidence of pseudopodia flattening. No evidence of hyaloplasm spreading between pseudopodia
**IV**	Spreading	Evidence of pseudopodia formation, with spreading of hyaloplasm
**V**	Fully-Spread	Fully-spread hyaloplasm with no distinct pseudopodia. Central bodies adopt a flattened state

For morphometric analysis of the surface-activated platelets, the polymer discs were retained following removal of the supernatant, and prepared for SEM. This was done by gently washing the discs with PBS, and then fixing any adhered platelets with 4% PFA for 20min, followed by dehydration with ascending concentrations of ethanol (20, 40, 60, 80, 100% w/v), with 20min of incubation for each step. This method of fixation and dehydration protects platelets from desiccation while concurrently reducing unwanted artefacts, thereby preserving platelet integrity for high resolution morphological analysis under SEM. The samples were air-dried in the laminar flow hood for 16–24hrs prior to SEM imaging.

### Data analysis

Parametric data has been expressed as mean ± standard deviation. SPSS 21.0 (IBM) was used to perform one-way ANOVA with post-hoc Bonferonni’s test for analysis of statistical significance. *p* <0.05 was considered statistically significant.

## Results

### Surface modifications after anti-CD34 antibody immobilization

**Figure 2:**
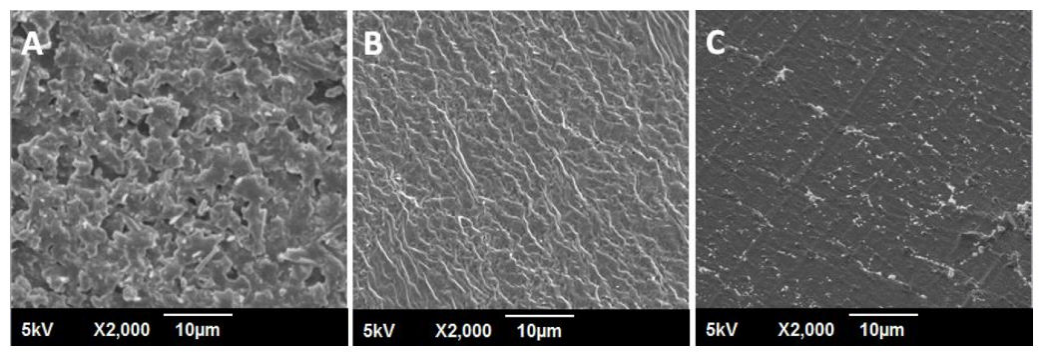
Surface characterization of unmodified and modified POSS-PCU surfaces by scanning electron microscopy at 2000x magnification. **A** POSS-PCU B POSS-PCU-FS C POSS-PCU-FS+CD34. POSS-PCU-FS: POSS-PCU with fumed silica anchors, POSS-PCU-FS+CD34: POSS-PCU biofunctionalized with anti-CD34 antibodies.

POSS-PCU displayed a rough and textured surface (Figure 2). This is most likely due to POSS nanocages migrating to the top of the surface after solvent evaporation [37]. POSS-PCU-FS displayed a ridge-like appearance on its surface. Upon anti-CD antibody immobilization, the ridges and rough textures are no longer visible, but instead the surface takes on a more uniform topography with globular structures. These globular structures could be due to protein aggregation attributed to the antibodies.

### Reduction in water contact angle

**Figure 3:**
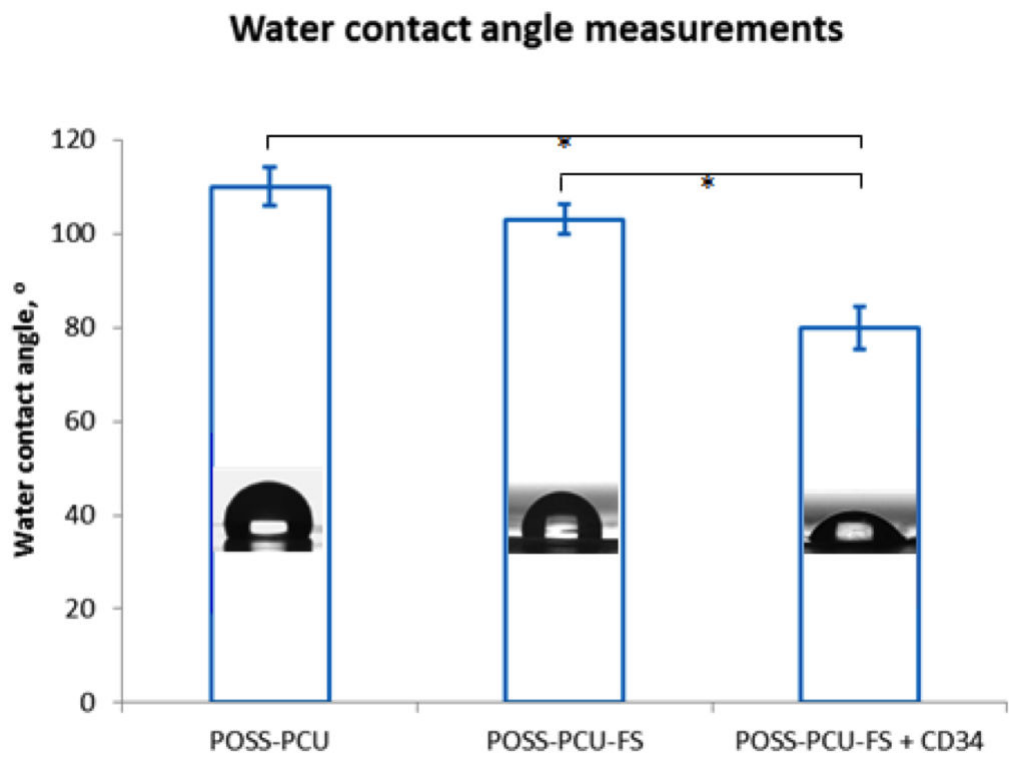
Water contact angle measurements of unmodified and modified POSS-PCU surfaces, using a sessile drop method. Biofunctionalization with anti-CD34 antibodies significantly reduces the mean water contact angle value compared to POSS-PCU and POSS-PCU-FS. Error bars: ± SD; * denotes *p* < 0.05. POSS-PCU-FS: POSS-PCU with fumed silica anchors, POSS-PCU-FS+CD34: POSS-PCU biofunctionalized with anti-CD34 antibodies.

POSS-PCU had a water contact angle of 110.0° ± 4.1°. POSS-PCU-FS had a slightly reduced water contact angle of 103.1° ± 3.2°. Conjugation with anti-CD34 antibodies resulted in a decreased water contact angle of 80.0° ± 4.5°(*p* < 0.05) (Figure 3).

### Stability of surface immobilization of antibodies

**Figure 4:**
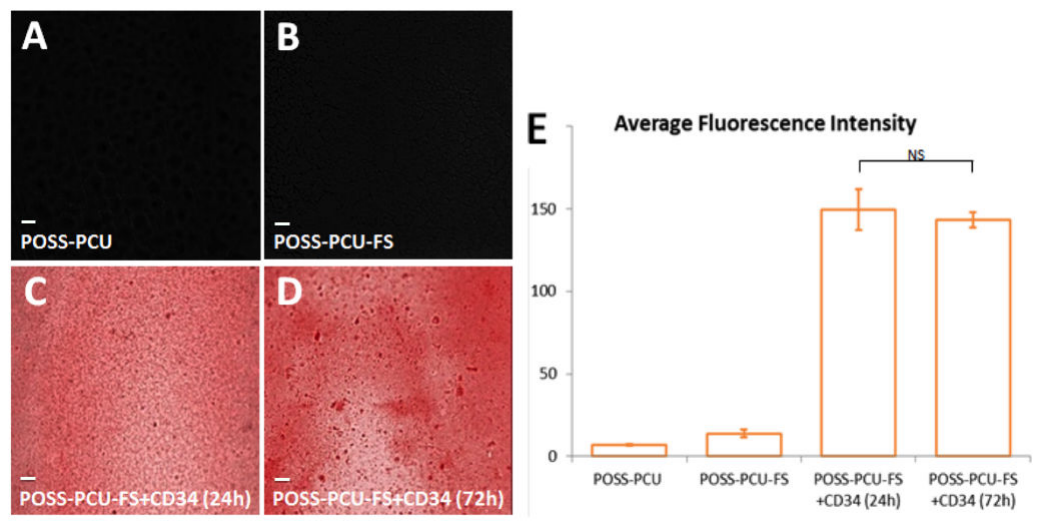
Detection of surface-immobilized anti-CD34 antibodies using quantum dots. When films were immunostained with red QD-IgG fluorescent labels, (A) control POSS-PCU and (B) POSS-PCU-FS did not exhibit fluorescence, as compared to (C-D) POSS-PCU-FS+CD34 films, which showed uniform immobilization of bound antibodies on the film even after washing by mechanical shaking for 24 and 72 hrs (10x magnification). (E) Measurements of samples’ residual fluorescent intensities after 24 and 72 hrs of washing demonstrates that antibodies remain stably bound. Error bars: ± SD; NS denotes no significant difference (*p* > 0.05). POSS-PCU-FS: POSS-PCU with fumed silica anchors, POSS-PCU-FS+CD34: POSS-PCU biofunctionalized with anti-CD34 antibodies.

Immunostaining using red QD-anti-mouse IgG (secondary antibody) confirmed the presence of anti-CD34 antibodies (primary antibody) on the polymer surface even after 24 and 72 hours of continuous washing. Fluorescence imaging revealed a uniform distribution of anti-CD34 antibodies. Results indicated the stability of antibody coating, without significant reduction in fluorescence intensity after 72 hours (149.8 ± 12.4 vs 143.4 ± 4.5, *p* > 0.05) (Figure 4).

### EPC extraction and characterization

**Figure 5:**
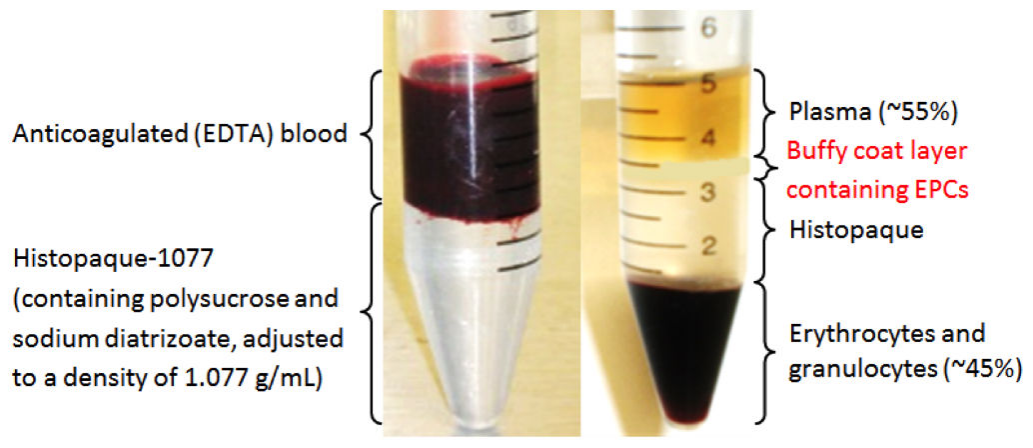
EPC extraction from peripheral blood. The buffy coat layer contains EPCs, which were cultured on test samples.

**Figure 6:**
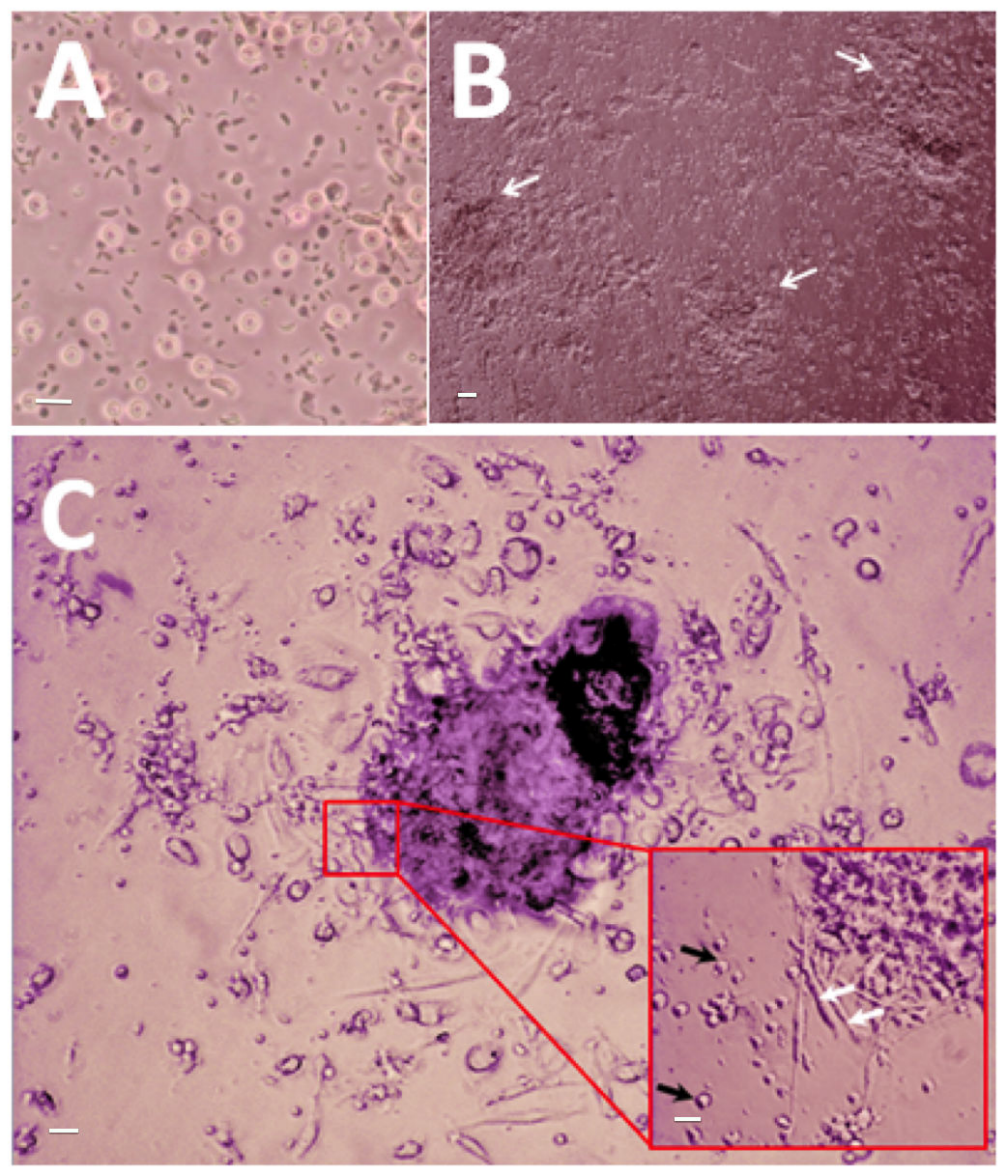
Morphological changes of human endothelial progenitor cells (EPCs) isolated from peripheral blood, and cultured for 7 days. (A) Peripheral blood mononuclear cells (PBMCs) containing a heterogenous population of EPCs, monocytes, and granulophages, plated on normal uncoated tissue culture plate on day 1 (40x magnification). (B) White arrows point towards EPC colonies observed at day 7 (10x magnification). (C) An EPC colony at day 7, defined morphologically as central cluster of rounded cells surrounded by multiple spindle-shaped cells (20x magnification). Inset: Black arrows point to multipotent stem cells; white arrows point to EPCs (40x magnification). Scale bar represents 20 µm.

6.32 × 10^7^ ± 2.94 × 10^6^ PBMCs were isolated from 20 ml of venous blood in with a protocol that was described previously [18,28,31]. This method allows the extraction of putative EPCs that would differentiate into cells with characteristic EC markers (Figure 5). Cells were fixed after 7 days, and were assessed to see if they had attained an EC-like phenotype. EPCs displayed elongated spindle-shaped morphology after 24 hours. At day 5, cell clusters began forming. At day 7, EPC clusters were seen as clusters of round cells surrounded by spindle-shaped cells along the periphery (Figure 6).

### Capturing EPCs with anti-CD34 antibody

**Figure 7:**
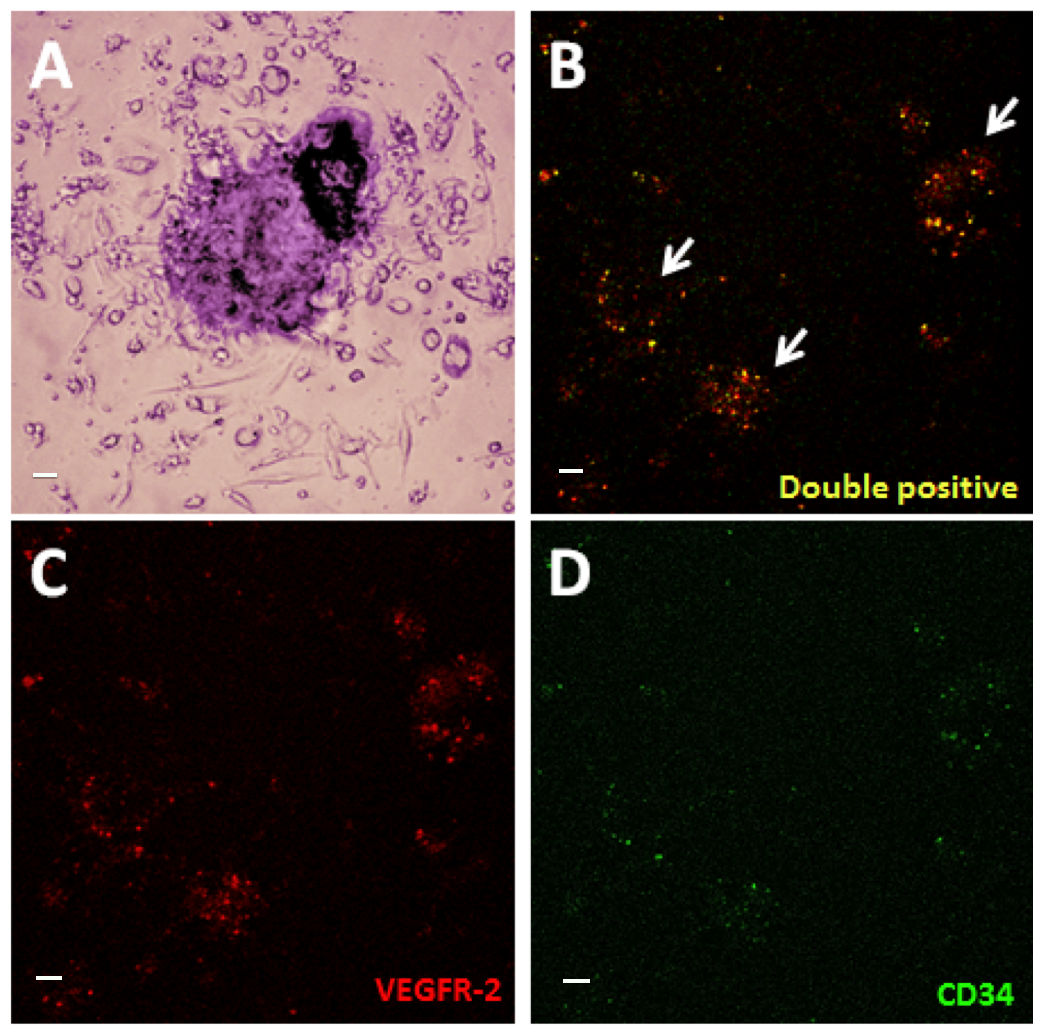
EPCs under light microscopy. (**A**) An EPC colony, defined morphologically as a central cluster of rounded cells surrounded by multiple spindle-shaped cells (20x magnification). (**B**-**D**) Expression of VEGFR-2 (red) and CD34 (green) was assessed under laser scanning confocal microscopy (10x magnification). Double-positive colonies (yellow) were identified as EPC colonies. Scale bar represents 20 µm.

**Figure 8:**
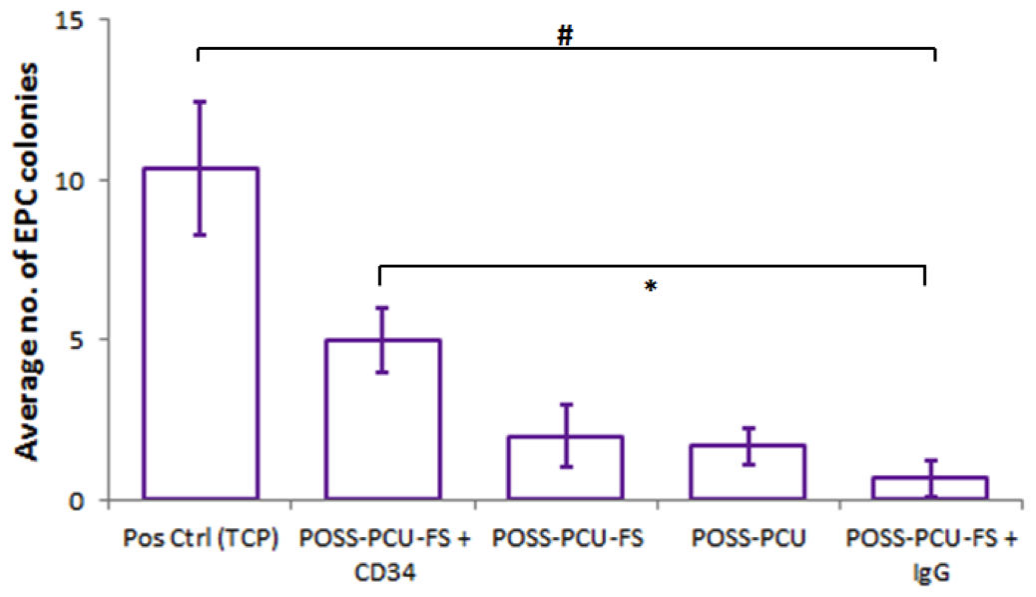
EPC colony counts. Number of double-positive CD34^+^ / VEGFR-2^+^ EPC colonies formed per 10^6^ cells plated was enumerated after 7 days of culture on the different surfaces. Error bars: ± SD; # denotes a significant difference (*p* < 0.05) between positive control and all other groups; * denotes a significant difference (*p* < 0.05) between POSS-PCU-FS+CD34 and POSS-PCU-FS+IgG. Scale bar represents 20 µm. POSS-PCU-FS: POSS-PCU with fumed silica anchors, POSS-PCU-FS+CD34: POSS-PCU biofunctionalized with anti-CD34 antibodies.

**Figure 9:**
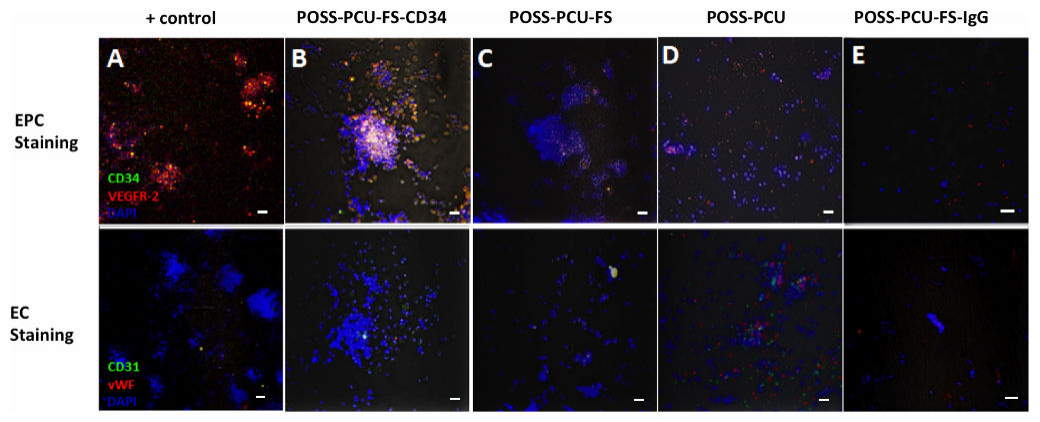
EPC capture assay. Cells were double-stained at day 7 for either EPC (CD34/VEGFR-2) or EC (CD31/vWF) markers, and counterstained with DAPI for nuclei. (**A**) Positive control, (**B**) POSS-PCU-FS+CD34, (**C**) POSS-PCU-FS, (**D**) POSS-PCU, (**E**) POSS-PCU-FS+IgG isotype negative control (20x magnification). Scale bar represents 20 µm. POSS-PCU-FS: POSS-PCU with fumed silica anchors, POSS-PCU-FS+CD34: POSS-PCU biofunctionalized with anti-CD34 antibodies, POSS-PCU-FS+IgG: POSS-PCU biofunctionalized with goat anti-mouse antibodies.

Growth of isolated PBMCs on the various polymer samples (POSS-PCU, POSS-PCU-FS, POSS-PCU-FS+CD34, POSS-PCU-FS+IgG) was monitored over a period of 7 days. Colonies were identified by QD-immunostaining for EPC markers (CD34/VEGFR-2) (Figure 7). The ability of these EPCs to proliferate and differentiate into an EC phenotype was further investigated by QD-immunostaining for EC markers (CD32/vWF). Generally, there was a lower number of EPC colonies for POSS-PCU-FS+IgG, POSS-PCU, and POSS-PCU-FS, compared to POSS-PCU-FS+CD34. The difference in colonies was statistically significant for POSS-PCU-FS+CD34 compared to POSS-PCU-FS-IgG (5.0 ± 1.0 vs 0.67± 0.58, *p* < 0.05) (Figure 8). No statistical significance was observed between POSS-PCU, POSS-PCU-FS, and POSS-PCU-FS+CD34 (*p* > 0.05). EPC colony numbers on positive control (10.3 ± 2.1) was higher and statistically significant compared to all other groups (*p* < 0.05). DAPI staining revealed that POSS-PCU-FS and POSS-PCU-FS+CD34 had a higher density of adhered cell clusters compared to POSS-PCU and POSS-PCU-FS+IgG. This high density of cell clusters appeared to be similar to positive control. Immunostaining for EPC markers showed that a small clusters of POSS-PCU-FS+CD34 were double-positive for CD34^+^/VEGFR-2^+^ (Figure 9). None of the samples were double positive for CD31^+^/vWF^+^.

### Observation of functional clotting kinetics

**Figure 10:**
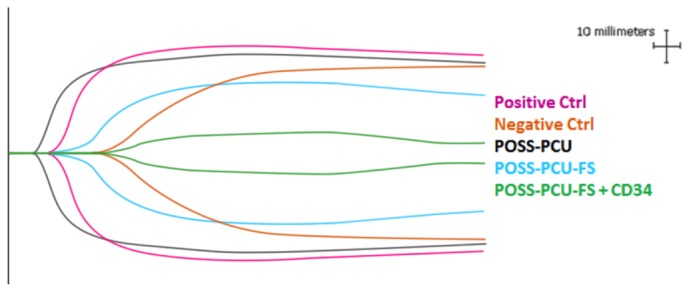
Representative TEG profiles of blood in contact with the modified and unmodified POSS-PCU surfaces. Standard unmodified TEG cups with citrated whole blood were used as positive controls, whereas standard cups containing citrated blood mixed with the anti-coagulant L-arginine were used as negative controls. Profiles demonstrate a similar cigar-shape, indicating functional hemostasis. POSS-PCU-FS: POSS-PCU with fumed silica anchors, POSS-PCU-FS+CD34: POSS-PCU biofunctionalized with anti-CD34 antibodies.

TEG was used to assess the degree of contact activation of the coagulation cascade on the various surfaces of polymer samples. All test groups demonstrated a typical cigar-shaped TEG profile, indicating functional coagulation in normal hemostasis (Figure 10).

**Figure 11:**
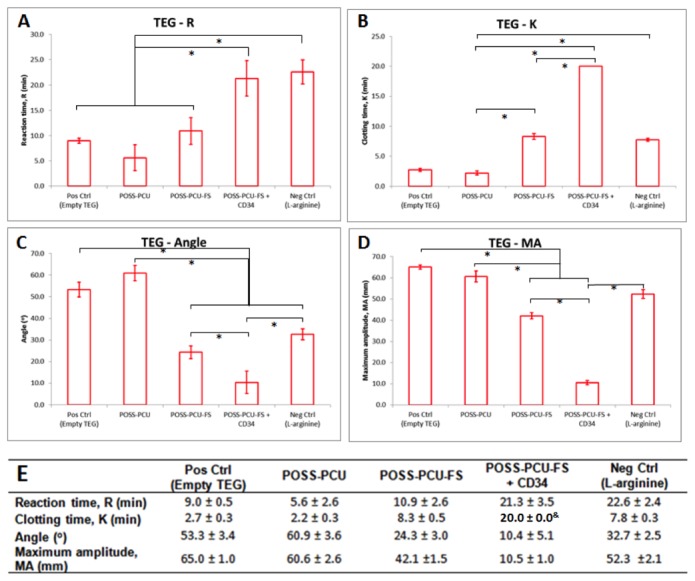
TEG parameters. Samples differed in (**A**) reaction time, (**B** and **C**) clotting rate, and (**D**) maximum clot strength. The mean of each clotting parameter was plotted (n=3). Error bars: ± SD; *denotes *p* < 0.05. (**E**) Table representing mean TEG parameters. ^&^Anti-CD34-coated samples never reached 20 mm clot size after 1.5 hrs of testing, hence k value was set as 20 min for ease of statistical testing. Standard unmodified TEG cups with citrated whole blood were used as positive controls, whereas standard-coated cups containing citrated blood mixed with the anti-coagulant L-arginine were used as negative controls. POSS-PCU-FS: POSS-PCU with fumed silica anchors, POSS-PCU-FS+CD34: POSS-PCU biofunctionalized with anti-CD34 antibodies.

TEG profile for POSS-PCU-FS+CD34 was statistically significant compared to other samples across all assessment parameters (*r*-time, *k*-time, α-angle, MA). POSS-PCU-FS+CD34 had an r-time of 21.3 ± 3.5. This was statistically significant (*p* < 0.05) from positive control, POSS-PCU, and POSS-PCU-FS, which had *r*-time of 9.0 ± 0.5, 5.6 ± 2.6, and 10.9 ± 2.6 respectively. *r*-time for POSS-PCU-FS+CD34 was not statistically significant (*p* > 0.05) compared to negative control (22.6 ± 2.4). POSS-PCU-FS+CD34 had a *k*-time of 20.0 ± 0.0. This was statistically significant (*p* < 0.05) from positive control, POSS-PCU, POSS-PCU-FS, and negative control which had *k*-time of 2.7 ± 0.3, 2.2 ± 0.3, 8.3 ± 0.5, and 7.8 ± 0.3 respectively. The α-angle for POSS-PCU-FS-CD34 was 10.4 ± 5.1. This was statistically significant (*p* < 0.05) compared to positive control, POSS-PCU, POSS-PCU-FS, and negative control, which had α-angles of 53.3 ± 3.4, 60.9 ± 3.6, 24.3 ± 3.0, and 32.7 ± 2.5 respectively. The MA for POSS-PCU-FS+CD34 was 10.5 ± 1.0. This was statistically significant from positive control, POSS-PCU, POSS-PCU-FS, and negative control, which had MA values of 65.0 ± 1.0, 60.6 ± 2.6, 42.1 ± 1.5, and 52.3 ± 2.1 respectively (Figure 11).

#### Reduced platelet activation and adhesion

Platelet activation can be characterized by a stark change in cell morphology that would upregulate adhesion and pro-coagulant factors.

**Figure 12:**
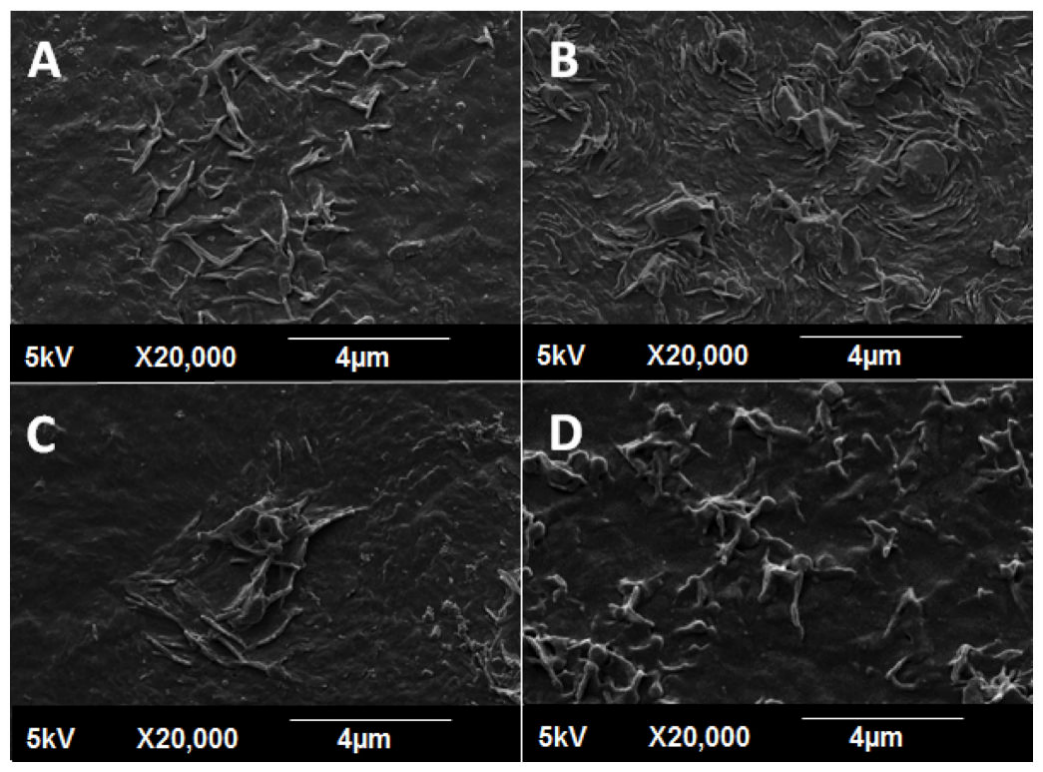
Platelet activation assay. SEM images (2000x magnification) of adhered platelets show distinctly different morphological appearances. (**A**) Collagen, (**B**) POSS-PCU, (**C**) POSS-PCU-FS, (**D**) POSS-PCU-FS+CD34. Adhered platelets found on collagen-coated surfaces (positive control, **A**), showed the highest degree of activation, with formation of distinct pseudopodia and hyaloplasm spreading. Platelets adhering on POSS-PCU (**B**) and POSS-PCU-FS (**C**) were mostly dendritic-spread, with prominent pseudopodia as well as some flattening. Those adhered to POSS-PCU-FS+CD34 (**D**) remained dendritic with a clear spherical body and without any evident flattening. POSS-PCU-FS: POSS-PCU with fumed silica anchors, POSS-PCU-FS+CD34: POSS-PCU biofunctionalized with anti-CD34 antibodies.

Culture plates coated with collagen displayed the greatest degree of platelet activation, with extensive spreading of hyaloplasm and flattening of central bodies, resembling the stage V morphology (Figure 12). Morphology of platelets on POSS-PCU and POSS-PCU-FS resembled between stage III and IV. They displayed a dendritic appearance with prominent pseudopodia projecting from platelet bodies, and also flattening and hyaloplasm spreading. In contrast, the appearance of platelets on POSS-PCU-FS+CD34 fit stage II, showing a rounded morphology with early pseudopodia formation, without any flattening.

**Figure 13:**
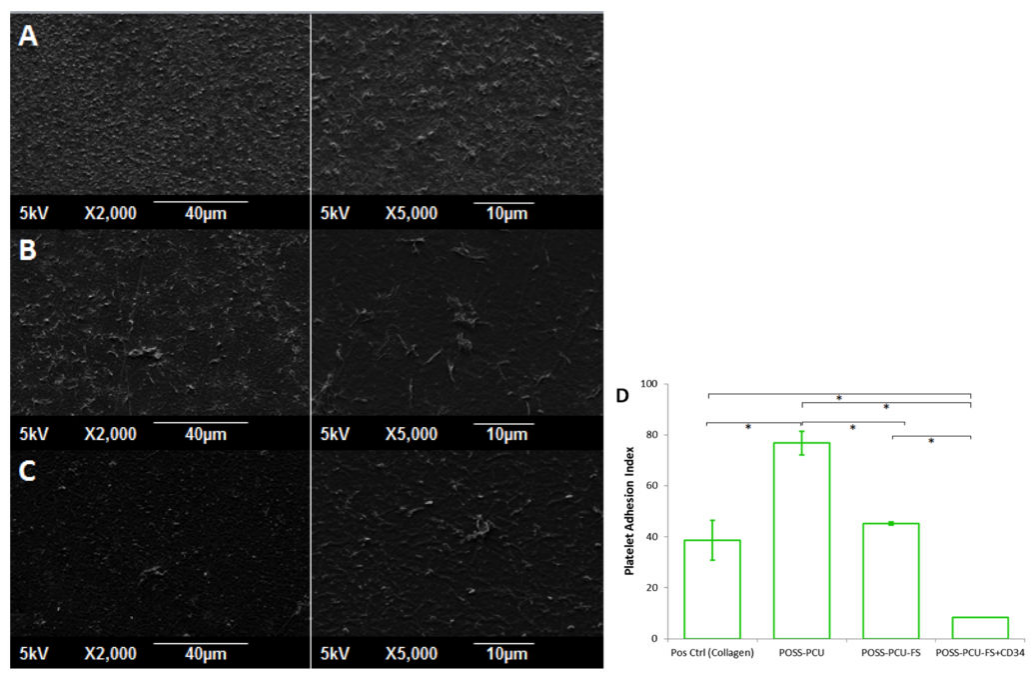
Platelet adhesion assay. (**A**-**C**) SEM images (2000x and 5000x magnification) show the greatest number of platelets adhering to POSS-PCU surfaces. (**A**) POSS-PCU, (**B**) POSS-PCU-FS, (**C**) POSS-PCU-FS+CD34. (**D**) The degree of platelet adhesion, expressed as the Platelet Adhesion Index, was significantly reduced by incorporation of FS and subsequent conjugation of anti-CD34 antibodies. *denotes a significant difference (*p* < 0.05). POSS-PCU-FS: POSS-PCU with fumed silica anchors, POSS-PCU-FS+CD34: POSS-PCU biofunctionalized with anti-CD34 antibodies.

Results of the platelet adhesion assay largely agrees with TEG and platelet activation assay. A high density of platelet adhesion on POSS-PCU was observed, which was corroborated by a high Platelet Adhesion Index (PAI), which was almost twice that seen in collagen (76.8 ± 7.8 vs 38.7 ± 14.0, *p* < 0.05) (Figure 13). PAI of POSS-PCU-FS was around half that of POSS-PCU (45.1 ± 4.6, *p* < 0.05). Immobilization of anti-CD34 antibodies further reduced the PAI to 8.4 ± 0.7 (*p* < 0.05).

## Discussion

The main premise of developing a biofuntionalized polymer for cardiovascular applications is to circumvent restenosis and thrombosis in order to sustain long-term patency and viability. Furthermore, adopting a “pro-healing” approach such as mobilizing endothelial progenitor cells to form a confluent layer of endothelium is one way to restore vessel homeostasis. POSS-PCU has been previously shown to function as a viable material for cardiovascular applications due to its superior biocompatibility and mechanical properties [21,38]. This study seeks to explore the concept of further biofunctionalizing POSS-PCU with anti-CD34 antibodies via FS-nano-anchors. Preliminary assessment of its EPC capturing efficacy and hemocompatibility was also evaluated.

The native vascular endothelium normally provides an efficient anti-thrombotic and anti-coagulant barrier against thrombus formation, lipid uptake, and inflammatory cell migration [6]. It also regulates VSMC growth by acting as a physical barrier separating VSMCs from circulating growth factors, and by secreting inhibitory factors that limit VSMC proliferation and migration [5]. It is hence not surprising that inevitable endothelial injury during PCI and the absence of an endothelial layer on stents are implicated in the restenotic and thrombotic events occurring post-PCI. The formation of a functional, competent endothelium on stent surfaces is therefore crucial to preventing both thrombosis and restenosis.

Early studies focussed on cultivating patient-isolated ECs to confluence on implant surfaces *in vitro*, before implanting the autologous endothelium back into the patient [39-41]. However, this strategy has several disadvantages that render it clinically unfeasible: firstly, an initial surgery is required for a vessel biopsy; secondly, retention of ECs on the implant surface under exposure to blood flow has proven to be a challenge; and thirdly, *in vitro* endothelialization is time-consuming and expensive, with a high risk of contamination [5,42]. An alternative strategy that has garnered much research attention in recent years involves capturing circulating endothelial progenitor cells (EPCs) onto the stent surface, thereby generating an autologous endothelium *in situ* [14,29].

EPCs are bone marrow-derived stem cells, which were first isolated from peripheral blood by Asahara et al. (1997) and shown to attain EC characteristics *in vitro* [43]. Although there is no agreed definition of EPCs or method for isolating EPCs at the time of writing, most studies agree that EPCs are characterised by the co-expression of CD34 and vascular endothelial growth factor receptor-2 (VEGFR-2), which are indicators of cell naïveté and a vascular EC phenotype respectively [44,45]. Additionally, these two cell surface antigens are common to embryonic EPCs and hematopoietic stem cells (HSCs). However, as both antigens are expressed on mature ECs as well, several studies have proposed CD133 as an additional indicator of cell naïveté to ensure EPC identity. Like CD34, CD133 expression is present in early HSCs, but is lost after differentiation [17,32,46,47].

EPCs have been widely investigated for tissue engineering applications for a number of reasons: firstly, their relative ease of isolation; secondly, their high capacity for proliferation *in vitro*; thirdly, their ability to retain a capacity for differentiation; and fourthly, their ability to migrate to sites of vascular injury. Numerous *in vitro* and *in vivo* studies have successfully demonstrated induction of EPC mobilisation, homing, incorporation to target sites, and/or differentiation into an EC monolayer. Notably, a substantial number of these studies employed surfaces biofunctionalized with the EPC-specific anti-CD34 antibody to capture circulating EPCs [14,18,28,48-54].

Encouraging results have been achieved with the anti-CD34-coated Genous^TM^ Stent (GS) (OrbusNeich), which follow-up studies have shown to be safe even after 3 years post-implantation [5,14,17,52,53,55]. The results of 3 clinical studies provide substantive evidence of significantly accelerated re-endothelialization of the stent surface and decreased thrombogenicity with the use of GS over BMS [53,55-58]. Anti-CD34-mediated EPC adhesion appeared to protect against the blood-implant interactions leading up to thrombosis and restenosis, such as the adhesion of platelets, fibrin, and inflammatory cells. Additional *in vitro* and *in vivo* experiments by Larsen et al. (2012) provide further support for anti-CD34 immobilization as a viable strategy for EPC capture [53]. An *in vitro* EPC-capture assay demonstrated a preferential adhesion of human peripheral blood-derived CD34^+^ cells to GS over BMS. An *in vivo* baboon AV shunt model showed that GS inhibits in-stent thrombosis, as indicated by a significantly lowered platelet deposition compared to BMS, while in a rabbit endothelial denudation model, analysis of EC markers expression after 7 days indicated that GS significantly promotes re-endothelialization [53].

Despite the large amount of literature on the EPC capture efficacy of anti-CD34-coated surfaces, much less work has been done on the hemocompatibility of these modified surfaces. While these surfaces aim to increase EPC adhesion, they inadvertently run the risk of increasing clotting tendencies by encouraging non-specific platelet or leukocyte adhesion [59]. It is therefore important for the anti-CD34-coated stent surface to be anti-thrombogenic and anti-inflammatory itself, so as not to activate pro-coagulatory cascades that jeopardize stent patency, and in the process, effectively address both issues of restenosis and thrombosis through rapid surface endothelialization *in situ*.

Immobilization of anti-CD34 antibodies via amine-functionalized FS and an EDC-NHS covalent linker proved to be a feasible method for obtaining a functional and stable surface for EPC capture. Distinct surface modifications were observed via SEM imaging, and a reduction of water contact angle was also seen after antibody immobilization.

Extensive cell adhesion and clustering was observed on POSS-PCU-FS+CD34 and POSS-PCU-FS after 7 days of culture, compared to POSS-PCU and POSS-PCU-FS+IgG. The higher degree of cell adhesion on POSS-PCU-FS compared to POSS-PCU could perhaps be explained by the positively charged amines on the surface of POSS-PCU-FS. The majority of the cells on POSS-PCU-FS+CD34 were CD34 positive, while none of the cells on non-specific IgG isotype negative control (POSS-PCU-FS+CD34) were CD34 positive. This indicates that immobilized anti-CD34 antibodies on POSS-PCU-FS+CD34 capture CD34^+^ cells via a specific binding, rather than a non-specific binding. However, the majority of cells captured on POSS-PCU-FS+CD34 were single-positive, being CD34^+^/VEGFR-2^-^ or CD34^-^/VEGFR-2^-^. The number of EPC colonies formed on POSS-PCU-FS+CD34 was significantly higher than POSS-PCU-FS-IgG (*p* < 0.05), but not POSS-PCU and POSS-PCU-FS (*p* > 0.05).

Literature on in vitro and in vivo studies indicate that CD34^+^ cells have the potential to differentiate into mature EC and VSMCs. Furthermore, EPCs can release growth factors such as VEGF, which can upregulate mobilization and proliferation of VSMCs [16,42]. Another important factor to consider is that EPCs make up a very small proportion of PBMCs; flow cytometry has indicated that only 0.002% of PBMCs are positive for CD34, and only 0.4 ± 0.2% of this percentage are EPCs, defined by being positive for VEGFR-2 and CD133 [16]. Evidence has shown that anti-CD34 antibodies can attract other cells such as granulocytes, monocytes, and plasma proteins like fibrinogen. Hence, there is a will be a possible scenario that these cells would also compete with CD34 positive EPCs for binding on anti-CD34 antibodies. There is also a distinct possibility that adherent cells on POSS-PCU-FS+CD34 would differentiate into other lineages such as VSMCs, rather than ECs. Interestingly enough, a study propounded the use of granulocyte colony stimulating factor (G-CSF) could mobilize EPC for the repair of injured arteries [60]. However, more in vivo studies would be needed to ascertain if the application of G-CSF in tandem with EPC-capturing stents could increase its endothelialization efficacy. It was previously reported that despite accelerated endothelialization on CD34-coated ePTFE grafts and stents, there was also a higher degree of intimal hyperplasia [61]. Hence, solely relying on anti-CD34 as a strategy to achieve endothelialization might not be sufficient, considering that there is a probability of indiscriminate binding of other cells to anti-CD34 antibodies. Indeed, there is a risk of intimal hyperplasia and increased cell proliferation index if cell capture technology is not sufficiently optimized for accuracy and specificity [5,29,30,52].

The inability of adhered EPCs differentiating into ECs could be explained by the relatively short culture period of 7 days. The static culture was not representative of cells within the luminal area of blood vessels, which are in 3D and exposed to flow conditions. Evidence suggests that cells grown in 3D perfused reactors might be more representative of physiological conditions compared to 2D static conditions. Furthermore, it has been shown that fluid shear stress can influence the differentiation of progenitor cells into endothelial cells [62]. Therefore, a dynamic flow circuit calibrated to physiological conditions might serve as a better approximation in studying the growth and proliferation of EPCs into ECs.

To ascertain the blood-compatibility of POSS-PCU and its antibody-immobilized version, contact activation and platelet assessments were carried out. TEG is used to assess coagulation kinetics in real-time, and is used in cardiac and transplant surgery. Therefore, TEG cuvettes were coated with the various test samples with the intention of seeing how blood reacts with them compared to uncoated cuvettes. POSS-PCU was observed to have the lowest reaction time (r-time: 5.6 ± 2.6 min) and clotting time (k-time: 2.2 ± 0.3 min) compared to POSS-PCU-FS and POSS-PCU-FS+CD34. This could be due to its hydrophobicity, reflected in its high water contact angle (110.0 ± 4.1). The relationship between hydrophobicity and thrombogenicity, however, is contentious. While there is evidence suggesting that hydrophobic materials such as ePTFE are thrombogenic due to its affinity for plasma proteins such as fibrinogen [59], others have argued the converse. It has also been postulated that due to the adsorption of different proteins on hydrophobic surfaces, there is increased competition between proteins and clotting factors, thereby reducing surface-induced activation of pro-coagulating factors [63]. Therefore, it seems likely that thrombogencity of a materials is often dictated by the type of proteins that are preferentially adsorbed onto the surface. For instance, fibrinogen would increase thrombogenicity, while albumin would decrease it [64-66]. More in-depth studies on the interaction of fibrinogen and POSS-PCU would therefore be crucial for understanding its hemocompatiblity.

POSS-PCU-FS displayed a longer clotting time (8.3 ± 0.5 vs 2.2 ±0.3, *p* < 0.05) and reaction time (10.9 ± 2.6 vs 5.6 ± 2.6, *p* > 0.05) compared to POSS-PCU. This may be due to its reduced water contact angle (103.1 ± 3.2 vs 110.0 ± 4.1, *p* > 0.05). Further surface characterization studies would be necessary to confirm this relationship. Upon immobilization with anti-CD34 antibodies, resulted in increased reaction time (21.3 ± 3.5 vs 10.9 ± 2.6, *p* < 0.05) and increased clotting time (20.0 ± 0.0 vs 8.3 ± 0.5, *p* < 0.05), when compared to POSS-PCU-FS. This apparent reduction in thrombogenicity and improved hemocompatibility may be due to modifications in surface biochemistry and nano-architecture. Three-dimensional globular structures can be seen on POSS-PCU-FS+CD34. This might contribute to surface passivation via steric repulsion of platelets and clotting factors [63].

Cigar-shaped plots were seen in TEG profiles, indicating that functional hemostasis had taken place without fibrinolysis. α-angle reflects the speed of the build-up of fibrin, giving an idea of clot formation. POSS-PCU had the highest speed of clot formation (60.6 ± 2.6), followed by POSS-PCU-FS (24.3 ± 3.0), and POSS-PCU-FS+CD34 (10.4 ± 5.1). MA is associated with the strength of clot, and indicates platelet-fibrin interactions. Similarly, POSS-PCU had the highest MA value (60.6 ± 2.6), followed by POSS-PCU-FS (24.1 ± 1.5), and POSS-PCU-FS+CD34 (10.5 ± 1.0).

Results of platelet activation and adhesion largely correlated with TEG profiles. It was interesting to note that POSS-PCU demonstrated the highest degree of platelet adhesion, even exceeding that of collagen. Although showing a relatively high degree of adhesion, SEM revealed that platelets were largely inactivated on POSS-PCU. Indeed, there is complex relationship between platelets and mobilization of EPCs, and evidence has shown that a certain degree of platelet adhesion and activation is needed for EPC growth and proliferation [67]. Hence, it could be extrapolated that POSS-PCU might be favorable for the growth and maintenance of EPCs into ECs in the long-term. Incorporation of amine-FS mitigated platelet adhesion, which could be due to a lower degree of hydrophobicity of the surface. Other studies have supported this observation of a lower platelet adhesion on charged hydrophilic surface, and vice versa [68,69]. Overall, it has been observed that POSS-PCU is rendered less thrombogenic by incorporation of amine-functionalized-FS. Thrombogenic response was further attenuated, with improved hemocompatiblity after anti-CD34 antibody conjugation.

Our future work regarding this aspect of biofunctionalized stent coatings would be to assess *in vitro* engineering mechanics, such as radial strength, percentrage recoil, and accelerated fatigue studies. *In vivo* studies would also have to be performed in animal models, and various studies like optical coherence tomography (OCT) to assess stent malapposition and the degree of re-endothelialization.

## Conclusion

We have demonstrated that clinical-grade POSS-PCU can be further bio-functionalized by attaching anti-CD34 antibodies on its surface. Anti-CD34 antibodies attached on POSS-PCU displayed increased cell adhesion, although it is likely that only a small sub-population of adhered cells were EPCs. It could be assumed that, although the vast majority of adhered cells were CD34^+^, their potential to differentiate into functional endothelial cells remain open to question. Nevertheless, this approach of actively attracting EPCs for in situ endothelialization on POSS-PCU increased its hemocompatiblity. It might also be useful to consider using various bioactive moieties, e.g. VEGFR-2, to be used in tandem with anti-CD34 for capturing EPCs as this will increase its specificity. It is also pertinent to accurately characterize the identity and functionality of the captured EPCs on the biomaterial surface, as this would be an imperative design consideration for its long-term viability. Taken together, this avenue of promoting *in situ* endothelialization and hemocompatibility warrants further refinement as it is of relevance in a plethora of cardiovascular applications.
